# Clinical Applications of Novel Tools to Personalize Follow-Up and Predict Outcomes in Congenital Heart Disease

**DOI:** 10.3390/jcm14020521

**Published:** 2025-01-15

**Authors:** Giorgia Grutter, Benedetta Leonardi

**Affiliations:** Pediatric Hospital Bambino Gesù, IRCCS, 00167 Rome, Italy; benedetta.leonardi@opbg.net

Recently, the application of novel tools to predict and personalize outcomes for congenital heart disease (CHD) patients has significantly transformed their treatment. The Special Issue “Clinical Applications of Novel Tools to Personalize the Follow-up and Predict Outcomes in Congenital Heart Disease” delves into innovative instrumental methods in CHD, emphasizing the role of automation, always guided by human oversight, in reducing human error by standardizing diagnoses and therapeutic approaches within defined limits. Artificial intelligence (AI) has demonstrated effectiveness in identifying patterns and anomalies within large datasets, with applications ranging from financial fraud detection to pinpointing irregularities in medical data. AI’s ability to process vast amounts of information efficiently has proven to be a valuable asset in medicine, especially in cardiology [1]. Early diagnosis is critical for congenital heart diseases, which involve a broad spectrum of structural and functional heart abnormalities [Contribution 1]. By processing extensive data quickly, AI provides clinicians with unparalleled insights into CHD’s complex anatomy and physiology. This capability allows for earlier and more accurate diagnoses, safer and more personalized treatment plans, and better long-term outcomes overall [Contributions 1–4]. Recently, the substantial potential of AI in leveraging cardiac imaging data for guiding treatment decisions and improving long-term outcomes has been demonstrated in patients with aortic valve disease [Contribution 3]. Aljassam et al. showed that AI can analyze aortic shape features preoperatively, aiding surgical decision-making and identifying subgroups with potentially poorer clinical outcomes postoperatively [Contribution 3]. Pre- and postoperative three-dimensional (3D) aortic models reconstructed from computed tomography (CT) and cardiac magnetic resonance (CMR) images of patients undergoing various types of aortic valve replacement (AVR), such as Ozaki, Ross, and valve-sparing procedures, were analyzed. Computational analyses for statistical shape modeling (SSM) and hierarchical clustering were performed separately for two subgroups, assessing the ascending aorta and the entire aorta to identify potential morphological classification variations related to shape inputs [Contribution 3]. Makan Rahshenas et al. developed a highly discriminative survival model based on the American College of Cardiology (ACC) classification for congenital heart disease [Contribution 2]. Their model demonstrated strong predictive capabilities for the 8-year survival of newborns with CHD, revealing significant survival rate differences among various ACC-CHD categories. The highest 8-year survival rate was 99.5% [0.989–0.997] for patients with interatrial communication abnormalities and ventricular septal defects, while the lowest was 34% [0.21–0.50] for patients with a functionally univentricular heart. Harrell’s C-index further confirmed the model’s discriminative ability, even when additional known predictors were included [Contribution 2]. Agorrody et al. highlighted the Kansas City Cardiomyopathy Questionnaire (KCCQ-12) as a promising tool for identifying patients with Fontan circulatory failure [Contribution 4]. When used alone or with other health-related quality of life (HRQOL) instruments, this tool provides valuable insights into patient outcomes and management strategies. Leonardi et al. emphasized the importance of novel tools in managing Tetralogy of Fallot patients [Contribution 5]. Abnormal myocardial T1 values on cardiac resonance imaging [2] and decreased peak oxygen consumption measured via cardiopulmonary testing [3] can help identify Tetralogy of Fallot patients at risk of adverse events, underscoring the need for these assessments in future clinical practice. Finally, Triantafyllou et al. underscored the significance of computed tomography angiography (CTA) in evaluating right-sided aortic arch (RAA) variants, offering critical insights for clinical practice [Contribution 6]. RAA of Type 1 (mirror-image) was found to have a pooled prevalence of 0.07%, while RAA of Type 2 (with unique arterial branching and a retroesophageal left subclavian artery) had a prevalence of <0.01%, indicating its rarity. Congenital heart anomalies, particularly cyanotic ones such as Tetralogy of Fallot, are strongly associated with Type 1 RAA, which appears in 75–85% of such cases. Conversely, Type 2 RAA is rarely linked to heart anomalies, except when coexisting with a left descending aorta.

## Summary and Future Directions

In summary, the studies presented in this Special Issue highlight the value of novel tools in managing patients with congenital heart disease. The insights gained emphasize the potential for personalized treatment and management strategies for high-risk patients, ultimately improving hemodynamic outcomes and overall prognosis. The integration of tools such as patient-reported outcome measures and advanced imaging techniques can enhance treatment safety and long-term sustainability. Further research is essential to validate these findings and refine the clinical application of these tools.
